# Acute Liver Failure among Patients on Efavirenz-Based Antiretroviral Therapy

**DOI:** 10.1155/2018/1270716

**Published:** 2018-05-10

**Authors:** Innocent Lule Segamwenge, Miriam Kaunanele Bernard

**Affiliations:** Department of Internal Medicine, Intermediate Hospital Oshakati, Oshakati, Namibia

## Abstract

**Objectives:**

To describe the clinical characteristics of patients presenting with fulminant liver failure after varying periods of exposure to Efavirenz containing antiretroviral medications.

**Methods:**

We report a series of 4 patients with human immunodeficiency virus (HIV) infection who were admitted with acute liver failure (ALF) over a 6-month period. All these patients had been treated with a range of Efavirenz containing antiretroviral regimens and were negative for hepatitis A, B, and C infections as well as other opportunistic infections, all were negative for autoimmune hepatitis, and none had evidence of chronic liver disease or use of alcohol or herbal medications. Information on patient clinical characteristics, current antiretroviral regimen, CD4 count, HIV-1 RNA levels, and clinical chemistry parameters was collected. Informed consent was provided.

**Results:**

During a 6-month period, four patients without other known risk factors for acute hepatitis presented with symptomatic drug-induced liver injury with varying symptoms and outcomes. The pattern of liver injury was hepatocellular for all the 4 cases. Liver biopsies were done for all the four cases and the results showed a heavy mixed inflammatory cell infiltrate with eosinophils. For three patients withdrawal of Efavirenz from their antiretroviral regimen was sufficient to restore transaminase levels to normal and led to improvement of clinical symptoms. For one patient his clinical course was characterized by fulminant liver failure and fluctuating episodes of hepatic encephalopathy which ultimately resulted in his death.

**Conclusion:**

Hepatotoxicity of Efavirenz is not as rare as previously described in the literature and does actually present with fatal outcomes. The key message to note is that frequent monitoring of liver enzymes should be done at initiation of antiretroviral therapy and should continue throughout the treatment period.

## 1. Introduction

Antiretroviral therapy (ART) has dramatically changed the life expectancy and natural course of HIV infection [1, 2]. These drugs however are lifelong and are not without side-effects. Long-term side-effects of highly active antiretroviral therapy now contribute significantly to the morbidity and mortality among HIV patients with good immune reconstitution.

There are several long-term and short-term toxicities of the different antiretroviral drugs affecting many organs in the body [3, 4]. Hepatotoxicity of antiretroviral drugs is one of the more serious and life-threatening complications of antiretroviral drugs.

Non-nucleoside reverse transcriptase inhibitors (NNRTIs) are the drugs most commonly implicated in hepatotoxicity. The two most commonly used NNRTIs Nevirapine and Efavirenz are frequently used as part of the triple combination first-line ART regimen [5]. Nevirapine use has been associated with significant hepatic injury compared to Efavirenz [6]. The toxicity of NNRTIs has also been found to be more common in the first 12 weeks of starting ART and also in patients coinfected with hepatitis B and C [6].

We describe a series of cases presenting with severe Efavirenz associated hepatocellular injury following long-term use of the drug, who were also negative for hepatitis B and C.

## 2. Case Presentation

### 2.1. Case 1

A 45-year-old female presented with fatigue and yellow discoloration of eyes and passing yellow urine for over 2 weeks. Her medical history was notable for a history on concurrent HIV infection diagnosed over 3 years previously and she had been on antiretroviral drugs for 1 year. Her antiretroviral regimen is comprised of Tenofovir Disoproxil Fumarate 300 mg (TDF), Lamivudine 300 mg, and Efavirenz 600 mg in a single pill combination tablet (TELURA®, Mylan).

Her CD4 count was 734 cells per mm^3^ and her viral load was below the level of detection at less than 40 copies of HIV per milliliter of blood (<40 copies/mL). She had no history of alcohol use nor any other drug use or chronic disease.

Her physical examination was notable for jaundice and tender right hypochondrium. She had no hepatic flap and no other features of hepatic encephalopathy or coagulopathy. The rest of her examination was normal.

Her liver function test showed a hepatocellular pattern of injury (Table 1). She was negative for hepatitis B and C. Her serology for autoimmune hepatitis which included antinuclear antibody (ANA), soluble liver antigen, anti-smooth muscle antibody, and liver kidney microsomal-1 antibodies was all negative. Her antiretroviral drugs were changed to Truvada (Tenofovir and Emtricitabine) and Raltegravir. A liver biopsy was done which showed acute hepatitis with interface activity and hydropic cytoplasmic changes with intermixed chronic inflammation with prominent eosinophil's and scanty neutrophils; these are findings which were more consistent with a drug-induced hepatic injury.

The antiretroviral drugs were all stopped and she was advised not to take any other potentially hepatotoxic drugs. During follow-up 2 months later, she was prescribed Tenofovir and Emtricitabine (Truvada) 300/600 mg once a day and Raltegravir (Isentress) 400 mg twice a day. Her ALT was 118 U/L (normal range 13–40 U/L), and gamma-glutamyl transferase (GGT) was 943 U/L (normal range 9–50 U/L). Her follow-up at 8 months, GGT was 227 U/L (normal range 9–50 U/L), and ALT and aspartate aminotransferase (AST) were within normal.

### 2.2. Case 2

A 40-year-old female presented with yellow discoloration of eyes, urine, and fluctuating level of consciousness over 1 week. Her medical history is noted for HIV infection diagnosed 4 months ago. She has since been on antiretroviral therapy consisting of Tenofovir Disoproxil Fumarate 300 mg (TDF), Lamivudine 300 mg (3TC), and Efavirenz 600 mg a single pill combination tablet (TELURA, Mylan). Her CD4 count was 402 cells per mm^3^ and her viral load was below level of detection at less than 40 copies per milliliter of blood (<40 copies/mL). She developed these symptoms 4 months after starting antiretroviral therapy.

She had no history of alcohol use or use of herbal remedies and supplements.

Her physical examination was notable for jaundice and presence hepatic flap; she had no bleeding diathesis and had grade 3 hepatic encephalopathy. Hepatitis screen A, B, and C and her serology for autoimmune hepatitis which included antinuclear antibody (ANA), soluble liver antigen, anti-smooth muscle antibody, and liver kidney microsomal-1 antibodies were all negative.

Her liver function showed hepatocellular pattern of injury shown in Table 1. Following the onset of the symptoms and signs above, her antiretroviral drugs were stopped at the time of presentation to hospital. A liver biopsy was also done which showed prominent inflammation of the portal tracts with infiltrates composed of neutrophils, lymphocytes, and several eosinophils; these are features consistent with medication induced liver injury. Her ALT at 1-month and 2-month follow-up were 42 and 37 U/L (normal range 13–40 U/L), respectively. The ALP was 161 and 105 U/L (normal range 40–120 U/L) at 1 and 2 months. All other blood tests were within normal limits. During her follow-up at 1 month, she was started on Abacavir, Lamivudine (Kivexa) 300/600 mg daily, and Dolutegravir (Tivicay) 50 mg daily. She has since recovered well.

### 2.3. Case 3

A 46-year-old male presented with yellow discoloration of his eyes and urine over for one month; his medical history was notable for HIV infection that was diagnosed 8 years ago. At the time of admission, he had been on antiretroviral therapy treatment for 8 years. The ART regimen consisted of Zidovudine (AZT), Lamivudine (3TC), and Nevirapine (NVP) (Cipla) a single tablet combination; however, 5 months prior to this presentation, his regimen was changed to Tenofovir Disoproxil Fumarate 300 mg (TDF), Lamivudine 300 mg, and Efavirenz 600 mg in a single pill combination tablet (TELURA, Mylan) (TDF, 3TC, and EFV) in May 2016. The CD4 count was 667 cells per mm^3^ and his viral load was below level of detection at less than 40 copies per millimeter of blood (<40 copies/ml). He had no history of drinking alcohol or use traditional herbal remedies.

The physical examination was notable for presence of jaundice, confusion, and excessive somnolence with presence of a hepatic flap. Tests for HBsAg, anti-HCV, anti-HAV IgM, CMV IgM, and EBV, antinuclear antibody (ANA), antimitochondrial antibody (AMA), and anti-smooth muscle antibody (ASMA) were negative. Abdominal ultrasound examination of the liver was normal. His liver function showed marked transaminitis as shown in Table 1. Liver biopsy was done which showed portal tracts with moderately heavy mixed inflammatory cell infiltrates of neutrophils, eosinophils, and lymphocytes. There were no plasma cells. The histological picture was in favor of drug-induced hepatitis.

His ART medications were stopped; however, his clinical course was characterized by recurrent episodes of hepatic encephalopathy without much improvement despite treatment with lactulose, metronidazole, and mannitol. His ALT was 339 U/L (normal range 13–40 U/L) at 1 month. He succumbed to fulminant liver failure after 1-month duration of hospitalization.

### 2.4. Case 4

A 41-year-old female presented with yellow discoloration of her eyes and urine over 3 weeks; she was diagnosed with HIV infection 9 years ago. She had been on ART treatment since her diagnosis. Her antiretroviral regimen consisted of Zidovudine (AZT) Lamivudine (3TC) and Nevirapine (NVP) (Cipla) a single tablet combination. Her CD4 count is 550 cells per mm^3^ and her viral load is below level of detection at less than 40 copies per millimeter of blood (<40 copies/ml). Her ART regimen was changed 2 weeks prior to presentation to Tenofovir Disoproxil Fumarate 300 mg (TDF), Lamivudine 300 mg, and Efavirenz 600 mg in a single pill combination tablet (TELURA, Mylan).

She had no history of alcohol or any other drug use. Her physical examination was notable for jaundice; there was no evidence of bleeding, no confusion, or other clinical signs of hepatic encephalopathy.

Tests for HBsAg, anti-HCV, anti-HAV IgM, CMV IgM, and EBV, antinuclear antibody (ANA), antimitochondrial antibody (AMA), and anti-smooth muscle antibody (ASMA) were negative. Her ALT above 942 U/L (normal range 13–40 U/L) and AST was above 913 U/L (normal range 10–40 U/L). Abdominal ultrasound examination of the liver was normal. Liver biopsy was done which showed normal hepatocytes with portal tracts having heavy mixed inflammatory cell infiltrates with numerous polymorphs and eosinophils. Cholestasis was evident with no granulomas, neoplasm, no fibrosis, or nodular formation.

Her ART was stopped and serial liver function testing was done to monitor her response to treatment. The ALT at weeks 2 and 4 of follow-up was 128 U/L and 114 U/l (normal range 13–40 U/L), respectively; the ALP was 153 and 185 U/L and GGT 250 and 310, respectively at weeks 2 and 4 of follow-up. She recovered well and at week two she was switched to a regimen consisting of Abacavir 300 mg twice daily, Lamivudine 150 mg twice daily, and Lopinavir boosted with ritonavir 400 m/100 mg twice daily. She recovered well.

## 3. Discussion 

Hepatotoxicity of antiretroviral drugs is one of the more serious and life-threatening complications of antiretroviral drugs. Non-nucleoside reverse transcriptase inhibitors (NNRTIs) are the drugs most commonly implicated in hepatotoxicity yet these drugs are frequently used as part of the triple combination first-line ART regimen [5]. The greatest risk of NNRTI-associated severe hepatotoxicity are observed in patients taking Nevirapine, those with hepatitis B or C coinfection, and those coadministered protease inhibitors [6]. In particular Nevirapine use has been associated with severe hepatotoxicity in other studies, which, in some cases, was associated with an early (12 weeks) hypersensitivity reaction. Likewise, Efavirenz has also been associated with significant hepatotoxicity; however severe hepatotoxicity is relatively uncommon among nonhepatitis C infected individuals and those not receiving protease inhibitor therapy [6].

Our patients presented with severe liver toxicity defined as grade 3-4 elevations (>5 × upper limit of normal) of aminotransferases AST or ALT but were negative for Hepatitis A, B, and C and were not on protease inhibitor therapy, and none was taking Nevirapine. Interestingly, cases 3 and 4 developed severe hepatotoxicity after switching from Nevirapine to Efavirenz. Case 3 developed drug-induced hepatitis after 5 months of changing to Efavirenz-based therapy while case 4 developed the same after only 2 weeks of switching therapy. Sulkowski et al. described the development of de novo severe hepatotoxicity among 7 of 85 (9%) Efavirenz users after changing from Nevirapine [6]. The mechanism underlying the development of this severe toxicity is not known; it is possible that patients may be susceptible to toxicity of a particular drug, rather than NNRTI class, specifically.

The average age was 43 years and all had high CD4 counts with the lowest being 402 and the highest 734 cells per mm^3^. High CD4 cell recovery has been associated with severe hepatotoxicity; it is not clear whether this represents immune-mediated liver injury in the hepatotoxicity or medication adherence and drug exposure [7]. The liver biopsies of our patients showed inflammatory cell infiltrates that are comprised predominantly of eosinophils and neutrophils with some biopsies showing lymphocyte infiltrates; these findings suggest that hepatic injury may be immune-mediated in our patients.

All our patients presented with symptomatic liver failure with case 3 having fulminant liver failure with a fatal outcome. Studies looking at grade 3-4 [8] hepatotoxicity of ART reported that most patients were asymptomatic and no deaths were due to liver-related events [7, 9, 10]. Fulminant hepatic failure due to Efavirenz leading to death is rare. Only one death due to fulminant liver failure after starting Efavirenz-based ART has been reported in the literature and two case reports of Efavirenz induced liver failure which required liver transplantation with good outcomes have been reported [11–13].

The timing of hepatotoxicity varied a lot among our patients ranging from two weeks of exposure to Efavirenz for case four to one year for case one. Hepatotoxicity due to Efavirenz has been described to occur between 100 days and 168 days (14 to 24 weeks) [6, 14]. Early and late onset types of antiretroviral-associated hepatotoxicity have been described. The early occurring form (less than 12 weeks after initiation of therapy) frequently goes along with rash, eosinophilia, fever, and arthralgia and seems to be based on an immune-mediated mechanism. The late onset form (after more than 12 weeks of therapy) is supposed to rely on an intrinsic toxic of the drug [14]. Our patient (case 4) who presented with early onset type of hepatotoxicity had no rash, fever, arthralgia, or eosinophilia. However, all our patients had eosinophil inflammatory infiltrate on liver biopsy. It is possible that both immune-mediated mechanisms and intrinsic toxic effects of the Efavirenz all had a role in the mechanism of hepatotoxicity in our patients.

## 4. Conclusion

Hepatotoxicity of Efavirenz is not as rare as previously described in the literature and does actually present with fatal outcomes. The key message to note is that frequent monitoring of liver enzymes should be done at initiation of antiretroviral therapy and should continue throughout the treatment period.

## Figures and Tables

**Table 1:** Patient characteristics and laboratory test results.

	Case 1	Case 2	Case 3	Case 4
Age (years)	45	40	46	41
Sex	F	F	M	F
Duration of EFV ART	12 months	4 months	5 months	2 weeks
CD4 count	734	402	667	550
Bilirubin total	208	291	471	273
Direct bilirubin	178	156	257	204
AST (NR: 10–40 U/L)	1288	377	913	913
ALT (NR: 13–40 U/L)	776	249	942	942
ALP (NR: 40–120 U/L)	229	143	139	338
GGT (NR: 9–50 U/L)	1101	145	337	735
R ratio	10.17	5.22	20.33	8.36
Hepatic damage type	Hepatocellular	Hepatocellular	Hepatocellular	Hepatocellular
RUCAM score	6	6	3	8

ALP: alkaline phosphatase; ALT: alanine aminotransferase; AST: aspartate aminotransferase; GGT: gamma-glutamyl transferase; NR: normal range; R ratio: (ALT/upper limit of normal range)/(ALP/upper limit of normal range); RUCAM: Roussel Uclaf Causality Assessment Method, ART: antiretroviral therapy, EFV: Efavirenz.
